# Erlotinib induces the human non–small‐cell lung cancer cells apoptosis via activating ROS‐dependent JNK pathways

**DOI:** 10.1002/cam4.881

**Published:** 2016-10-10

**Authors:** Fenglian Shan, Zewei Shao, Shenghua Jiang, Zhaozhong Cheng

**Affiliations:** ^1^ Medical CollegeQingdao UniversityQingdaoShandong266021China; ^2^ Affilitated Hospital of Jining Medical UniversityJiningShandong272000China; ^3^ Jining Medical UniversityJiningShandong272000China

**Keywords:** Apoptosis, EFGR, erlotinib, JNK, MMP, ROS

## Abstract

Although erlotinib (ERL) has drawn more and more attention toward its anticancer properties effect, the underlying mechanisms of ERL's anticancer properties effect remain unclear yet. So, the aim of this research was to explore the underlying anticancer mechanisms of ERL and to explore whether the reactive oxygen species (ROS)‐dependent c‐Jun N‐terminal kinase (JNK) pathway contributed to the anticancer properties provided by ERL. In our study, we used MTT assay to detect the anticell growth ability of ERL on human non–small‐cell lung cancer cell lines (A549). The extent of cell apoptosis was determined by Hoechst 33342 staining and fluorescence‐activated cell sorter (FACS) assay. Then, DCFH‐DA and JC‐1 staining were used to monitor intracellular reactive oxygen species (ROS) and mitochondrial membrane potential (MMP), respectively. Finally, the effect of ERL on phosphorylation state of JNK protein and downstream apoptosis concerned proteins were detected by western blotting assay. Results showed that ERL significantly suppressed the growth and reproduction of A549 cells with the concentration rising up in vitro. Hoechst 33342 staining and FACS assay also confirmed the proapoptosis effect of ERL on A549 cells with the concentration rising up. Furthermore, exposure of A549 cells to ERL increased the intracellular ROS production. As expected, intracellular ROS activated the proapoptotic JNK signaling pathway and inhibited the activation of EFGR signaling pathway. Our results also revealed that ERL could induce cell‐cycle arrest at G0/G1 period. Activation of JNK protein decreased MMP and downregulated content of antiapoptotic protein Bcl‐2 concomitant with the upregulated content of proapoptotic protein Bax in A549 cells. In addition, c‐Jun and cleaved caspase‐3 were also activated by the phosphorylated JNK induced by ERL. All of these proapoptosis effect of ERL was reversed by administration of N‐acetylcysteine (NAC), which performed as a ROS scavenger. Our results suggest that ERL induces A549 cells apoptosis via activating ROS‐dependent JNK pathways in human non–small lung cancer cells that provide a new experimental foundation for cancer therapy.

## Introduction

Lung cancer is believed to be one of the most common cause of cancer‐related deaths worldwide. Approximately 85–90% of all lung cancer deaths were resulted by non–small‐cell lung cancer (NSCLC) 1. Currently, growing researches have been performed to exploring effective strategies and drugs in order to ameliorate or cure cancer damage to the body. Lots of studies have confirmed that chemotherapy remains to be one of the most effective methods in the treatment of cancer because the patients' quality of life was greatly improved and life expectancy was effectively prolonged 2. Agents that are against multiple molecular targets are urgently needed for the treatment of lung cancer, including NSCLC 3.

**Figure 1:**
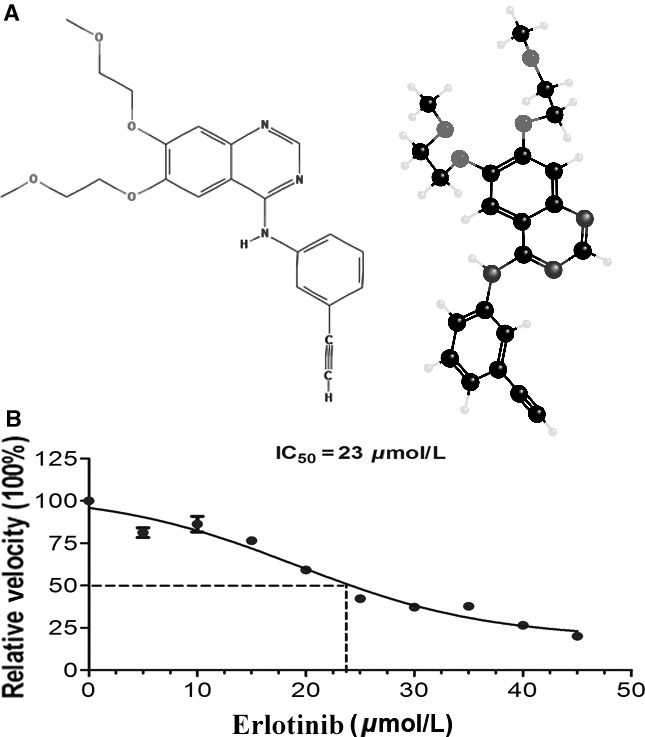
Effects of ERL on cell growth inhibition study. (A) The chemical structure of ERL. (B) Human non–small‐cell lung cancer A549 cells were treated with various concentrations of ERL (0, 5, 10, 15, 20, 25, 30, 35, 40, and 45 *μ*mol/L) and cultured for 24 h. Cell viabilities were measured by MTT assay. The IC‐50 value of ERL was appropriately at 23* μ*mol/L. ERL, erlotinib.

A newer and novel class of anticancer drug was the tyrosine kinase inhibitor which receives more and more attention from both clinical and basic research. Erlotinib hydrochloride (ERL, Fig. 1A), a U.S. Food and Drug Administration‐approved drug, approved initially for NSCLC, is an epidermal growth factor receptor (EGFR) tyrosine kinase (TK) inhibitor. The EGFR is one of the member of the erbB family of receptor tyrosine kinase proteins, which were also composed of several important members, including human epidermal growth factor receptor (HER)2/neu(erbB2), HER3(erbB3), and HER4 (erbB4). Interaction with ligand can phosphorylate the receptor TK phosphorylation and activate downstream intracellular signaling pathways, including the ras‐raf‐mitogen‐activated protein kinase (MAPK) and phosphatidylinositol 3 kinase–protein kinase (PK) B/Akt pathways, which play vital roles in regulating cellular proliferation and other survival activities 4. Research on multiple tumors showed that the expression of intracellular EGFR enhanced significantly, including breast, head and neck, bladder cancers, and NSCLC. Intracellular high levels of EGFR and/or HER2 have proved that it was related with a poor prognosis of many cancers, particularly in patients with NSCLC 5. ERL binds to the adenosine triphosphate (ATP) binding site of the EGFR via competing with ligand ATP in a reversible way. When ERL binds to the EGFR's extracellular domain, the receptor dimerization took place, which further autophosphorylates the critical tyrosine residues on the cytoplasmic terminal implicated in cell proliferation and the survival of cancers. Previous studies have reported that many signaling pathways are involved in ERL‐induced anticancer effect, such as p27^KIP1^ expression in human NSCLC cell growth 6. Therefore, the precise and molecular mechanisms involved in ERL‐induced human NSCLC cell apoptosis need to be elucidated urgently.

Apoptosis represents the dominant cause of programmed cell death, which plays a vital role in controlling the growth and development of living organisms 2. One of the crucial strategies for prevention and treatment of cancer was apoptosis induction. After all, the ultimate aim of chemotherapy is to successfully eliminate cancer cells 7. It is generally known that the survival or death of a cell depends on the balance between prosurvival and proapoptosis protein‐related signaling pathways 8. Reactive oxygen species (ROS) combined with biochemical antioxidants normally exist in the above balance to regulate cell life activities including development, growth, survival, and death 9. Moreover, the excess ROS production and/or antioxidant depletion can disrupt this critical balance and oxidative stress may occur in normal healthy cells 10. ROS play their destruction effects and then finally lead to pathological effects or alter their physiological action through modification of intracellular or extracellular macromolecules, and hyper‐ or hypofunctionality of the correlation signaling pathways 11. An increase in ROS can next activate the redox sensitive c‐Jun N‐terminal kinase (JNK) signaling pathway, which plays a vital role in activating mitochondrial concern apoptotic pathways 12. More and more evidence revealed that compared to normal healthy cells, cancer cells can produce more ROS which make them vulnerable to chemotherapeutic agents that further increases the production of ROS or reduces antioxidant defense of cells 13. It has been confirmed that induced oxidant stress can be a potential effective strategy selectively toxic toward cancer cells exerted by many chemotherapeutic drugs. Thus, ROS has been recognized as an important target for the development of anticancer drugs 14.

In our present study, we first detected that whether ERL induced the cell growth inhibition and cell apoptosis in human lung cancer A549 cell lines. In order to further investigate the antitumor mechanism of ERL, we measured the levels of intracellular ROS, mitochondrial membrane potential (MMP), p‐EFGR expression, and JNK signaling pathway, which are commonly believed to be associated with the apoptosis signal transduction pathway and affect the chemosensitivity of tumor cells to anticancer agents. This research proves that ERL induces the human NSCLC cells apoptosis via activating ROS‐dependent JNK signaling pathway.

## Methods

### Reagents and antibodies

N‐acetylcysteine (NAC), Hoechst 33342 stain, 3‐(4, 5‐dimethyl‐2‐thiazolyl)‐2, 5‐diphenyl‐2‐H‐tetrazolium bromide (MTT), 5, 5′, 6, 6′‐tetrachloro‐1, 1′, 3, 3′‐tetraethyl‐imidacarbocyanine iodide (JC‐1), and carbonyl cyanide 3‐chlorophenylhydrazone (CCCP) were purchased from Sigma‐Aldrich (St. Louis, USA). ERL was purchased from Sigma Chemicals and dissolved in dimethyl sulfoxide (DMSO) to produce a stock solution. A working solution was diluted from the stock solution using cell culture medium. All antibodies were purchased from (Sigma‐Aldrich, St. Louis, USA).

### Cell lines and cell culture

The human lung cancer A549 cells were purchased from Cell Bank of Shanghai Institute of Biochemistry and Cell Biology, Chinese Academy of Sciences (Shanghai, China). Cells were cultured in Dulbecco's modified Eagle's medium (DMEM) (Gibco, Carlsbad, CA, USA) supplemented with 10% fetal bovine serum (Gibco) at 37°C in a humidified atmosphere of 5% CO_2_.

### Cell growth inhibition studies of ERL

ERL (Fig. 1A) is a small molecule and reversible inhibitor of EGFR. It is widely used for the treatment of NSCLC and pancreatic cancer currently in clinical therapy. We first examined the IC‐50 values of the ERL on the A594 cells by fluorescence‐activated cell sorter (FACS, BD Biosciences, USA) method. Cells were treated with various concentrations of ERL (0, 5, 10, 15, 20, 25, 30, 35, 40, and 45 *μ*mol/L) and cultured for 24 h. After exposure to different concentrations of drugs, cell viability was detected by FACS on several groups of cells. The obtained results were expressed by relative velocity (100%) compared with no ERL‐treated cell group.

### Hoechst staining

Human lung cancer A549 cells were seeded in 96‐well culture plates and treated with the indicated concentrations of ERL (0, 5, 15, 25, 35, and 45 *μ*mol/L) for 24 h. After treatment, cells were stained with Hoechst 33342 at 37°C for 20 min in the dark, washed with PBS, and observed by fluorescence‐inverted microscopy (IX73; Olympus, Tokyo, Japan).

### Measurement of intracellular ROS

DCFH‐DA, as one of the membrane‐permeable probe, was used to detect the formation of ROS in cell lines. The nonfluorescent dye freely penetrates cells and is then hydrolyzed by intracellular esterase to DCFH and trapped inside the cells. Intracellular H_2_O_2_ or low‐molecular‐weight peroxides oxidize DCFH to the highly fluorescent compound DCF.

Following treatment with final concentrations of ERL (0, 5, 15, 25, 35, and 45 *μ*mol/L) for 24 h, cells were incubated with DCFH‐DA dye (50 *μ*mol/L, final concentration) in medium for 30 min in the dark. After rinsed twice with PBS solution, lysis buffer was added into dishes to break membrane of cells. Cell suspensions were transferred to 96‐well plate, and fluorescence was read at the Ex of 490 nm and the Em of 520 nm.

### Detection of MMP

Changes in MMP were measured with JC‐1 staining. Human lung cancer A549 cells were treated with final concentrations of ERL (0, 5, 15, 25, 35, and 45 *μ*mol/L) for 24 h. The collected cells were stained with JC‐1 working solution (10 *μ*g/mL) at 37°C in the dark for 20 min. The transfected cells were analyzed by a flow cytometer (Becton Dickinson, USA). The cells treated with CCCP (10 *μ*mol/L) for 20 min were used as the positive control group.

### Analysis of the effect of ERL on apoptosis and cell‐cycle arrest

We chose 0 and 25 *μ*mol/L as control and ERL groups, respectively. The antioxidant NAC (1 mmol/L) or no NAC were applied at the same time with 0 or 25 *μ*mol/L ERL on both groups cells treatment for 24 h. Then, the cells were stained by Annexin V‐APC in conjunction with propidium iodide (PI) and assessed by FACS method to determine the apoptosis of A549 cells. At the same time, cell‐cycle distribution was assessed by FACS on both NAC (1 mmol/L) and no NAC groups.

### Western blot analysis

The expression quantity of various proteins was determined in human lung cancer A549 cells using western blotting according to standard procedures. In short, total protein from untreated or treated cells was extracted in RIPA lysis buffer. The same amount of protein (30 *μ*g) from each group were separated with sodium dodecyl sulfate–polyacrylamide gel electrophoresis and transferred onto a PVDF membrane (Bio‐Rad Laboratories, Hercules, USA). Each membrane was incubated with a specific primary antibody (1:1000) at 4°C overnight after blocking with 5% skim milk at room temperature for 1 h. After three washes with washing buffer (20 mmol/L Tris‐HCl, 500 mmol/L NaCl, and 0.1% Tween 20), each suitable secondary antibody was incubated in the membrane at room temperature for 2 h. An ECL Advanced Western Blot Detection Kit (Thermo Fisher, Waltham, USA) was used to visualize the specific protein bands.

### Statistical analysis

Data are expressed as the mean ± standard deviation (SD) of triplicate samples. Statistical analysis for multiple comparisons was analyzed by a one‐way analysis of variance (ANOVA). *P* values below 0.05 were considered to be statistically significant.

## Results

### The proapoptosis effect of ERL on A549 cells

A549 lung cancer cells were incubated with various concentrations of ERL (0, 5, 10, 15, 20, 25, 30, 35, 40, and 45 *μ*mol/L) for 24 h. As shown in Figure 1B, ERL significantly increased the death of A549 cells. The higher concentration of the ERL, the stronger apoptosis effect emerged on A549 cells. According to the FACS results, we determined the IC‐50 values of the ERL in the A549 cells, which was approximately 23 *μ*mol/L. On the basis of IC‐50 values, we used 25 *μ*mol/L as ERL standard treatment concentration.

### The effect of ERL on A549 cells by Hoechst 33342 staining

**Figure 2:**
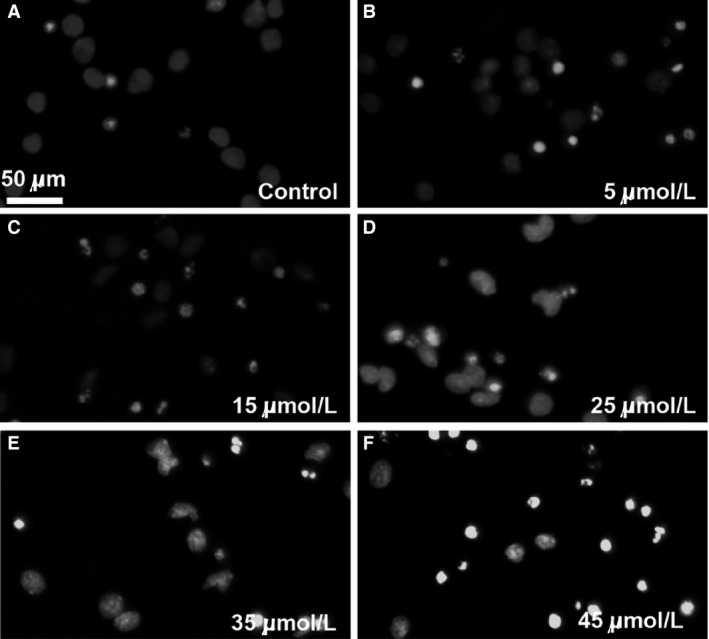
ERL‐induced apoptosis in A549 cells (A–F). Cells were treated with various concentrations of ERL (0, 5, 15, 25, 35, and 45 *μ*mol/L) for 24 h and the nuclei were stained by Hoechst 33342. ERL, erlotinib.

In order to further investigate the mechanisms about the ERL on A549 cell, we chose ERL at 0, 5, 15, 25, 35, and 45 *μ*mol/L in our study. As shown in Figure 2, after treatment with ERL 24 h, A549 cells stained with Hoechst 33342 indicate apoptotic morphological characteristics. A549 cells in control group showed regular and round nuclei as observed under the microscope (Fig. 2A). The cells' nuclei were observed for condensation and fragmentation after exposed to ERL for 24 h, which was recognized as characteristic of apoptotic cells (Fig. 2B–F). In conclusion, the result suggested that ERL could promote cell apoptosis with the treatment concentration rising up.

### Effects of ERL on the levels of ROS in lung cancer A549 cells

**Figure 3:**
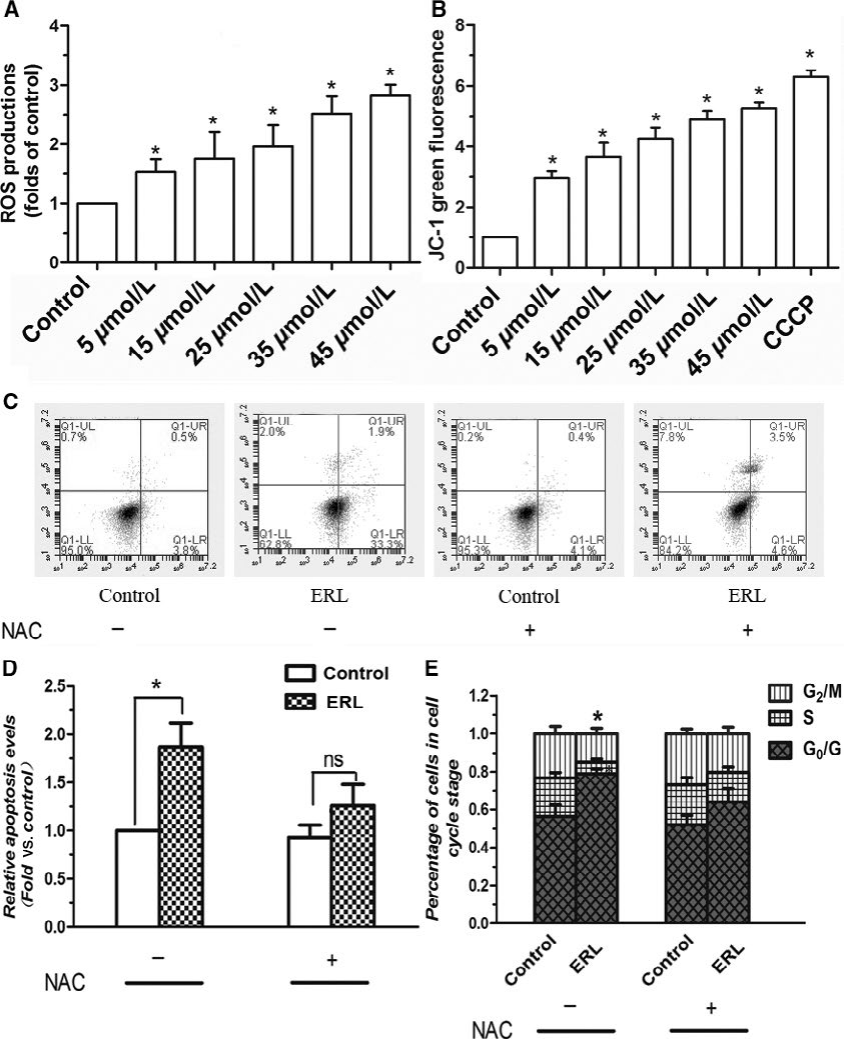
Effects of ERL on apoptosis, ROS, MMP, and cell‐cycle arrest test in A549 cells. (A) Cells were treated with various concentrations of ERL (0, 5, 15, 25, 35, and 45 *μ*mol/L) markedly increased ROS generation. (B) Cells were treated with various concentrations of ERL (0, 5, 15, 25, 35,45 *μ*mol/L), and the MMP was determined by flow cytometer using JC‐1 staining. (C, D) The effect of NAC on ERL‐induced cell apoptosis. (E) The effect of NAC on ERL‐induced cell‐cycle arrest assay. Data were presented as mean ± SD. *P < 0.05 relative to control group, ns: no significant differences. ERL, erlotinib; MMP, mitochondrial membrane potential; NAC, N‐acetylcysteine; ROS, reactive oxygen species.

To determine whether treatment of cells with ERL is associated with the generation of ROS, A549 cells were incubated with DCFH‐DA. As shown in Figure 3A, in A549 cells, the quantity of ROS increased significantly after treated with various concentrations of ERL for 24 h (*P* < 0.05), suggesting that the ERL induced the generation of ROS in A549 cells. The higher concentration of the ERL, the stronger generation of ROS emerged on A549 cells. These data conclusively suggested that ERL‐induced A549 cells apoptosis tightly related with the level changes of ROS in human lung cancer A549 cells.

### Effects of ERL on the MMP in A549 cells

It has been demonstrated that decreased MMP is one of the main characteristics of early apoptosis. Therefore, to investigate whether mitochondria were involved in ERL‐induced apoptosis, we measured MMP in A549 cells by flow cytometry using JC‐1 staining. As shown in Figure 3B, A549 cells treated with ERL showed a significant loss of MMP compared with control group. The higher concentration of the ERL, the stronger decreasing of MMP emerged on A549 cells. As expected, the positive control treatment CCCP (10 *μ*mol/L) resulted in a significant decrease of MMP in A549 cells.

### Underlying mechanism of ERL‐induced cell apoptosis and cell‐cycle arrest

In order to further confirm the effect of ROS in cell apoptosis, we put the ROS scavenger (NAC) in our study. As we expected, the proapoptosis effect of ERL was obviously reversed by the administration of NAC, Figure 3C and D. This result confirmed that ERL‐induced the generation of ROS played a critical role in the antitumor effect of ERL in A549 cells.

Next, we analyzed the effect of ERL on the cell‐cycle progression by FACS. Treatment of ERL induced a marked G0/G1 arrest in A549 cells (*P* < 0.05, Fig. 3E). Similarly, treatment with NAC could reverse the ERL's cell‐cycle arrest effect. Combined with previous results, we confirmed that the generation of ROS was a key factor in the proapoptosis effect of ERL.

### Effects of ERL on the activation of p‐JNK in A549 cells

**Figure 4:**
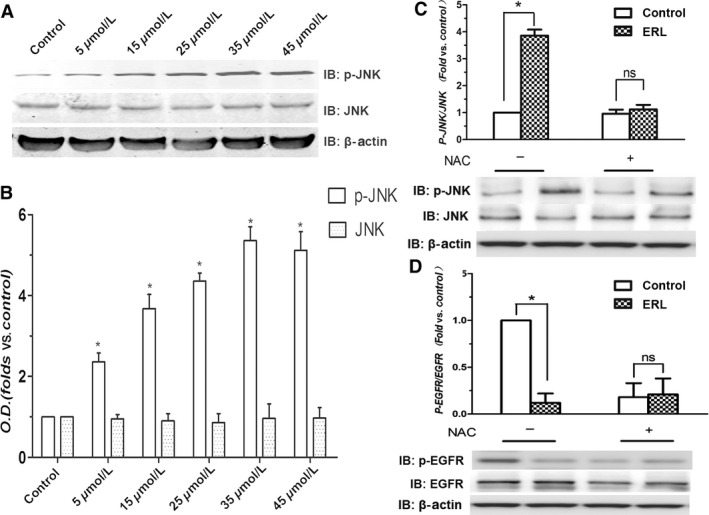
Effects of ERL on the phosphorylation of JNK and EGFR in A549 cells. (A) A549 cells were treated with various concentrations of ERL (0, 5, 15, 25, 35, and 45 *μ*mol/L), and the expressions of total and phosphorylated JNK were detected by western blot. (B) The intensity of the bands was expressed as optical density (OD) analysis. (C) The effect of NAC on ERL‐induced the expression ratio of p‐JNK/JNK. (D) The effect of NAC on ERL‐induced changes on the ratio of p‐EGFR/EGFR. Data were presented as mean ± SD. *P < 0.05 relative to control group, ns: no significant differences. EGFR, epidermal growth factor receptor; ERL, erlotinib; c‐Jun N‐terminal kinase; NAC, N‐acetylcysteine.

We wanted to verify whether the activation of the redox‐sensitive JNK signaling pathway was followed by an increase in ROS production. To investigate whether ERL‐induced ROS leads to the activation of JNK in A549 cells, we determined the expression of phosphorylation state of JNK in A549 cells treated with ERL. The results were shown in Figure 4A and B. ERL treatment for 24 h significantly increased the phosphorylation of JNK in A549 cells compared with the control group (*P *< 0.05). Besides, the overexpression of p‐JNK induced by ERL was significantly inhibited by the administration of NAC, Figure 4C. These data conclusively suggested that ERL‐induced the generation of ROS contributed to the activation of JNK signaling pathway in A549 cells.

### Effects of ERL on inhibition of p‐EGFR in A549 cells

As we all know, ERL is a one of the inhibitor of EGFR, so we next verified the effect of ERL on the activation of the protein EGFR. As is shown in the Figure 4D, the results showed that ERL significantly inhibited the expression of p‐EGFR compared with the control group (*P* < 0.05). Besides, the administration of NAC obviously reversed this effect of ERL. We may conclude that ERL exerts the direct connection with downregulation of EGFR signaling pathway which may have some potential relationship with the generation of ROS in A549 cells.

### Effects of ERL on the expression of apoptosis‐related protein

**Figure 5:**
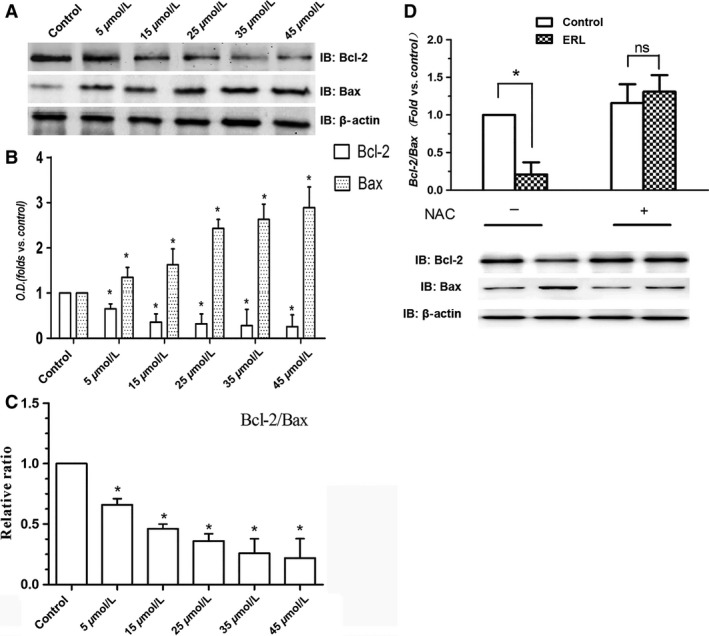
Effects of ERL on the expression of Bcl‐2 and Bax in A549 cells. (A) A549 cells were treated with various concentrations of ERL (0, 5, 15, 25, 35, and 45 *μ*mol/L), and the expressions of Bcl‐2 and Bax were detected by western blot. (B) The intensity of the bands was expressed as optical density (OD) analysis. (C) The relative ratio of Bcl‐2/Bax protein levels was reduced as A549 cells were coincubated with increasing concentrations of ERL. (D) The effect of NAC on ERL‐induced changes on the ratio of Bcl‐2/Bax. Data were presented as mean ± SD. *P < 0.05 relative to control group, ns: no significant differences. ERL, erlotinib; NAC, N‐acetylcysteine.

**Figure 6:**
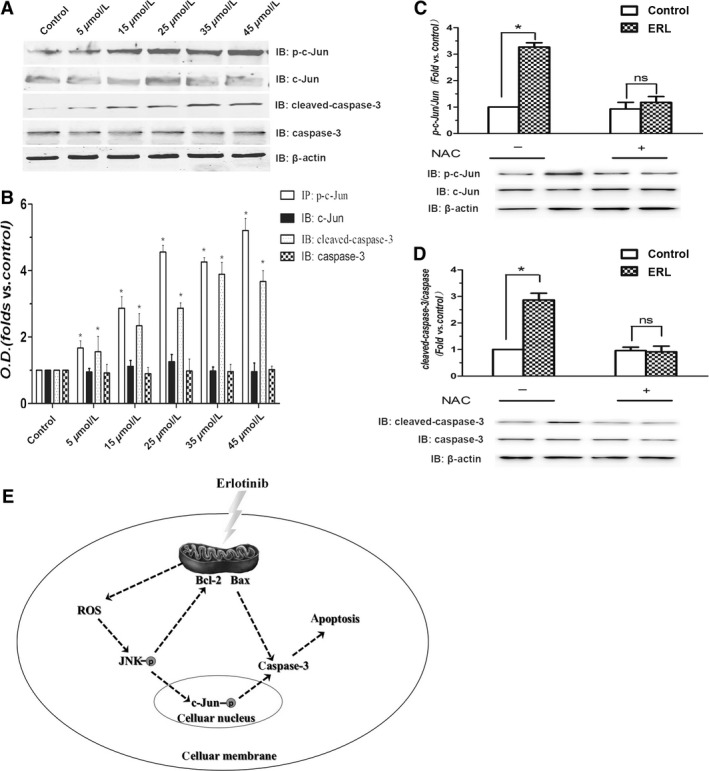
Effects of ERL on the phosphorylation of c‐Jun and the expression of cleaved caspase‐3 in A549 cells. (A) A549 cells were treated with various concentrations of ERL (0, 5, 15, 25, 35, and 45 *μ*mol/L), and the phosphorylation of c‐Jun and the protein expression of cleaved caspase‐3 were detected by western blot. (B) The intensity of the bands was expressed as optical density (OD) analysis. (C) The effect of NAC on ERL‐induced changes on the ratio of p‐c‐Jun/c‐Jun. (D) The effect of NAC on ERL‐induced changes on the ratio of cleaved caspase‐3/caspase‐3. Data were presented as mean ± SD. *P < 0.05 relative to control group, ns: no significant differences. (E) ERL could increase the level of ROS, which further activates JNK signaling pathway and then lead to A549 cells apoptosis. ERL, erlotinib; c‐Jun N‐terminal kinase; NAC, N‐acetylcysteine; ROS, reactive oxygen species.

Previous studies have demonstrated that c‐Jun is a nuclear substrate of JNK and the activated JNK, in turn, phosphorylates c‐Jun, which further activate those proteins associated with apoptosis such as Bcl‐2, Bax, and caspase‐3. Thereby, it is essential to address the effect of ERL on the phosphorylation of c‐Jun, and the expression of Bcl‐2, Bax, and caspase‐3. Figure 5 shows the results of western blotting with anti‐Bcl‐2 and anti‐Bax antibodies in A549 cells after ERL treatment for 24 h. Compared with the control group, the protein expression of Bcl‐2 significantly decreased and Bax increased in ERL treatment groups (Fig. 5A and B; *P* < 0.05). Moreover, the relative ratio of Bcl‐2/Bax protein also reduced in ERL treatment groups compared with the control group (Fig. 5C; *P* < 0.05). The administration of NAC obviously reversed this effect of ERL on the decreased ratio of Bcl/Bax. In addition, ERL treatment significantly increased the phosphorylation of c‐Jun and the expression of cleaved caspase‐3 in A549 cells compared with the control group (Fig. 6A and B; *P* < 0.05). Similarly with other apoptotic proteins, the administration of NAC obviously reversed the effect of activation of proapoptosis proteins of ERL (Fig. 6C and D).

## Discussion

ERL is believed to be one of the potential drugs for the treatment of cancer and this compound is in the clinical trials for lung cancer and lymphomas 15. Here, we provide solid evidence that ERL possesses definite anticancer effect in vitro. The proliferation of A549 cells was obviously inhibited by the administration of ERL and this profound effect was in a dose‐dependent manner. Apoptosis induction is one of the major effective pathways and most important property for candidate anticancer drug 16.

Apoptosis, a programmed cell death, is generated by many physiological and pathological conditions, and is considered to play a pivotal role in the development of cancer, including cancer initiation, progression, and metastasis 17, 18. Three different ways can initiate the cell apoptosis: the extrinsic death receptor pathway, the intrinsic endoplasmic reticulum stress pathway, and the intrinsic mitochondrion‐dependent pathway 19. The permeabilization of the mitochondrial outer membrane which occurs in response to various stimuli is believed to be the key event of the intrinsic mitochondrion‐dependent pathway, and is regulated by many cytoplasmic proteins including family members of Bcl‐2 20. The proteins of the Bcl‐2 family are important regulators of apoptosis, including five antiapoptotic proteins (Bcl‐2, Bcl‐xL, Bcl‐w, A1, and Mcl1), and two groups of proapoptotic proteins (Bax and BH3) 17. Thus, the ratio of Bax/Bcl‐2, as candidate prognostic biomarkers for lung cancer, indicates the degree of mitochondrial outer membrane permeabilization and hence the entrance to the execution phase of the apoptotic program 21. Therefore, we analyzed the expression of two of these proteins (Bax and Bcl‐2) and the Bax/Bcl‐2 ratio in the present study. We found that when treated with ERL, a decrease in Bcl‐2 expression accompanied with concomitant increases in Bax protein expression in A549 cells. The excess activation of Bax cooperates with the decreasing expression of Bcl‐2 to increase mitochondrial membrane permeability and consequently releases proapoptotic molecules which consequently activates the executioner caspases (3, 6, and 7) leading to apoptosis 22, 23. Therefore, it is easy to explain our results about the increase in caspase‐3 and cleaved caspase‐3 expression observed in our study which further supports the role of ERL in activating the executioner caspases and in contributing to cancer cell apoptosis.

Many chemical and physiological phenomena that are capable of inducing apoptosis are known to provoke oxidative stress via the generation of excess ROS, which suggests a close relationship between oxidative stress and apoptosis 24. ROS are thought to participate in a wide variety of cellular functions, including cell proliferation, differentiation, and apoptosis 25. ROS generations are the by‐products of cellular oxidative processes and further induce depolarization of the mitochondrial membrane then consequently produce an increase in the levels of other proapoptotic molecules in the cells 26. ROS and oxidative stress are both known as two of apoptosis triggers and modulators. Emerging evidence indicates that high level of ROS is required for the initiation of apoptotic responses induced by several anticancer agents 27. The present findings provide direct evidence in the lung cancer cells that ERL enhances ROS generation, which is responsible for the proapoptotic effects of ERL on lung cancer cells. Figure 3A clearly showed that ERL induced apoptosis concomitant with increased ROS generation in A549 cells, indicating that ERL may exert anticancer activity by increasing the production of ROS.

Several studies have demonstrated that apoptotic cell death induced by ROS is mediated by the activation of MAPK pathways 28. MAPK pathways are one of the numerous downstream cascades of the ROS signaling pathway closely associated with cell proliferation, differentiation, mitosis, survival, and apoptosis 29. JNKs, members of MAPK family, are extremely important in the process of apoptotic cell death 30. A variety of intracellular responses, such as inflammation, cell‐cycle regulation, cell death, development, differentiation, senescence, and tumorigenesis can be affected by the JNK protein kinase; as such, the JNK protein kinases have been exploited for the development of therapeutics to treat a variety of different diseases, including cancer 31. For instance, genipin, an aglycone derived from geniposide, can inhibit proliferation and induce apoptosis of K562 cells induced by Fas ligand and JNK activation; and CMS‐9, separating phospholipase A2 isolated from *Naja nigricollis* venom, activates the JNK signaling pathway and stimulates mitochondrial apoptosis 32. Therefore, our further investigation on the underlying anticancer effects of ERL in A549 cells has great importance both in basic and clinical research. We found that ERK treatment dramatically increased p‐JNK and p‐c‐Jun levels. These results suggest that ERK induced apoptotic cell death to some extent through activating ROS‐dependent JNK signaling pathway (Fig. 6E). These results might provide some clues to understand the mechanism underlying anticancer and to find clinical therapies for NSCLC patients using ERL in the near future.

## Conflict of Interest

The authors declare no competing financial interests.
